# Propaganda: The Effort Towards Union

**Published:** 1918-03-30

**Authors:** 


### NURSING AFFAIRS IN IRELAND.

### Propaganda: The Effort Towards Union.

Whenever a man numbers amongst his possessions a religion in which he really believes it invariably happens that his paramount desire is to find converts. This principle, for such it is, is not confined to religion. The missionary spirit is a human attribute shared by the worst type of criminal. One of the most active fields of missionary work is the world of politics. Every political argument is a recruiting effort, and probably the most completely equipped machine of propaganda that the world has ever known was manufactured in Germany. No conceivable artifice has been forgotten or overlooked, and no corner of the world neglected.

Propaganda in the nursing world is in its infancy, and it is my desire to convince those who aim after union that if success is to be achieved the wholehearted service and enthusiasm of the rank and file must be enlisted. Occasional articles in the public and the professional press count for something. Public meetings count for something. But neither in your time nor mine will a Union of Nurses be formed by relying on such means. There is a vast army of nurses who are not newspaper readers, and who lack time, inclination, or opportunity for attending meetings. The average nurse is a hard-worked woman with little time for play, and in her off-time she likes to get away from "shop." She is right. Moreover, the enemy of union is awake, and is actively engaged by night and by day sowing tares. The garden of lies is well cultivated, and the seeds are borne by the wind which bloweth where it listeth.

### Only One Standard.

The College of Nursing represents the greatest and most successful effort toward union that has yet been made. I might go further and say that it is the only effort of its kind. There have been, and are, a variety of cults and cliques, but if they are carefully examined it will be found that they correspond exactly with sects in religion, where some trumpery fad takes precedence of Christianity in its essence. Mrs. A has one little school, Mrs. B another, and Mrs. A and Mrs. B make infinite effort to gather recruits to their standards. In the world of nursing there ought to be one only standard, the standard of Union. After all, what else matters? What else counts for anything real or substantial?

### The Completest Effort towards Union.

May I be permitted at this point, and purely in self-defence, to point out that I am in no respect, directly or indirectly, associated with the College of Nursing? I am not even prepared to allege that it is a perfect institution. Probably it is not. I have yet to meet with any perfect human institution. Neither am I prepared to say that our friends the factionmongers are without their good features. The one overwhelming fact that appeals to me in connection with the College is that it is an effort towards union, an effort magnificently conceived and far-reaching, gathering to its potential fold the hundreds of thousands of nurses in every corner of the world whose proud banner is the British flag. This, in the scope of a sentence, is my apologia, and, so far as I am concerned, fine points of doctrine do not count. I have been hammering the Irish Nurses' Association. The women who compose it are very few in number, and they are good women, worthy of personal reverence. But they belong to a day that is dead, and not a few of them have a maggot in their brain. Their Early Victorian ideas survive the march of time and progress. In a word, they have no sympathy with Union, in which undoubtedly lies the salvation of the nursing profession. This spirit of conservatism is probably a direct result of our insularity, and probably also of our humanity. In Arnold Bennett's play, "Milestones," every old man exclaims, when a new idea is presented to him, "The country is going to the dogs!" Our grandchildren will regard the best of us as fossils.

### The Voice of the Multitude.

The note to strike at the moment is Union, and every nursing newspaper ought to ring with it. Every nurse ought to read the story of the siege of Jericho by Joshua. With one voice they shouted, and the walls of the citadel fell. There was no Legislative Assembly; no Act of Parliament. There was one voice, the voice of a multitude, and that voice prevailed. For the ultimate triumph of freedom and civilisation I have more faith in the voice of the Allied nations than I have in their guns, and they have very good guns. The Hun may carry his victorious arms hither and thither, and he may enlist in his service all the armies of frightfulness and devilry. But he cannot win. The power that makes for righteousness must inevitably succeed.

### The Individual Nurse's Part.

One of the finest conceptions of our time is a Union of Nurses—*i.e.*, the College of Nursing. The idea is in its babyhood, but already there are eight thousand two hundred enrolled. Every one of these thousands ought at once to become a recruiting agent. Each should be asked, as a point of honour and as a proof of loyalty, to find one recruit. This is not a large order, nor a difficult one to execute. I venture to prophesy that if this idea is adopted with thoroughness and zeal the entire profession will before many weeks find themselves in a single camp. Think what this would mean, especially at a moment when the nurse has the nation's ear. It would mean bread-and-butter, real and metaphorical, to the profession, shorter hours of labour, more liberal remuneration, longer holidays, and something to look forward to when inevitable age and infirmity arrive.

Missionary work in the cause, I venture to suggest, is worth considering. IERNE.

